# Chronic chemotherapeutic stress promotes evolution of stemness and WNT/beta-catenin signaling in colorectal cancer cells: implications for clinical use of WNT-signaling inhibitors

**DOI:** 10.18632/oncotarget.3934

**Published:** 2015-05-11

**Authors:** Meriam Ayadi, Anaïs Bouygues, Djamila Ouaret, Nathalie Ferrand, Salem Chouaib, Jean-Paul Thiery, Christian Muchardt, Michèle Sabbah, Annette K Larsen

**Affiliations:** ^1^ Cancer Biology and Therapeutics, Centre de Recherche Saint-Antoine, Paris, France; ^2^ Institut National de la Santé et de la Recherche Médicale (INSERM), Paris, France; ^3^ Institut Universitaire de Cancérologie, Pierre et Marie Curie (UPMC) Sorbonne Universités, Paris, France; ^4^ Institut National de la Santé et de la Recherche Médicale (INSERM), Gustave-Roussy, Villejuif, France; ^5^ Institute of Molecular and Cell Biology, A*STAR (Agency for Science, Technology and Research), Singapore, Singapore; ^6^ Department of Biochemistry, School of Medicine, National University of Singapore, Singapore; ^7^ Cancer Science Institute, National University of Singapore, Singapore; ^8^ Laboratory of Epigenetic Regulation, Centre National de la Recherche Scientifique (CNRS) URA2578, Institut Pasteur, Paris, France

**Keywords:** colorectal cancer, chemotherapeutic stress, “stemness”, WNT/beta-catenin signaling, WNT inhibitors

## Abstract

Most solid tumors contain a subfraction of cells with stem/progenitor cell features. Stem cells are naturally chemoresistant suggesting that chronic chemotherapeutic stress may select for cells with increased “stemness”. We carried out a comprehensive molecular and functional analysis of six independently selected colorectal cancer (CRC) cell lines with acquired resistance to three different chemotherapeutic agents derived from two distinct parental cell lines. Chronic drug exposure resulted in complex alterations of stem cell markers that could be classified into three categories: 1) one cell line, HT-29/5-FU, showed increased “stemness” and WNT-signaling, 2) three cell lines showed decreased expression of stem cell markers, decreased aldehyde dehydrogenase activity, attenuated WNT-signaling and lost the capacity to form colonospheres and 3) two cell lines displayed prominent expression of ABC transporters with a heterogeneous response for stem cell markers. While WNT-signaling could be attenuated in the HT-29/5-FU cells by the WNT-signaling inhibitors ICG-001 and PKF-118, this was not accompanied by any selective growth inhibitory effect suggesting that the cytotoxic activity of these compounds is not directly linked to WNT-signaling inhibition. We conclude that classical WNT-signaling inhibitors have toxic off-target activities that need to be addressed for clinical development.

## INTRODUCTION

Increasing experimental and clinical evidence indicates that tumors evolve over time which is driven by clonal heterogeneity, selection, non-genetic instability and adaptation to environmental stress like exposure to therapeutic agents [1–7]. Most studies of acquired drug-resistance have focused on classical mechanisms directly linked to the drug-target interaction like membrane transporters, drug distribution or DNA repair mechanisms, sometimes classified as “non-oncogenic” resistance [8–12]. However, recent findings suggest that chronic chemotherapeutic stress may promote more general and often unexpected biological changes in cancer cells. For example, analysis of biopsies from non-small cell lung cancer (NSCLC) patients with acquired resistance to small molecule epidermal growth factor receptor (EGFR) inhibitors displayed *MET* gene amplifications, epithelial to mesenchymal transition (EMT) as well as a transformation from NSCLC into small cell lung cancer [13]. Exposure of colorectal cancer (CRC) cells to oxaliplatin was associated with up-regulation of VEGF ligands and receptors [14] while chronic exposure to irinotecan was accompanied by activation of EGFR- and SRC-signaling in CRC models [15].

Most, if not all, solid tumors contain a subpopulation that displays molecular and functional similarities to stem cells. Stem cells are naturally chemoresistant due to high expression of certain ATP-binding cassette transporters, proficient DNA repair, a slow cell cycle and activation of various signaling pathways including WNT [16]. Therefore, one potential mechanism of acquired resistance to chemotherapeutic stress would be selection of cells with increased “stemness”.

The WNT-signaling pathway plays an important role in the colon. Starting at the base of the crypt unit, WNT-signaling is highest in the stem cell compartment and decreases as the cell moves upwards through the proliferative areas and into the differentiative compartment [17]. The central role of WNT signaling is further emphasized by a recent molecular study of human colon and rectal cancers indicating that at least 90% of patient tumors display a deregulated WNT-signaling pathway [2]. These findings support a role for WNT/beta-catenin-signaling inhibitors as potential novel agents for treatment of CRC. Assuming that acquired chemoresistance is accompanied by increased “stemness” and upregulation of WNT-signaling, such inhibitors might show preferential activity toward tumors with acquired drug resistance.

To establish the influence of chronic chemotherapeutic stress on “stemness”, we carried out a comprehensive molecular and functional analysis of six independently selected CRC cell lines with acquired resistance to three chemotherapeutic agents with different mechanisms of action (5-fluorouracil, oxaliplatin and irinotecan) derived from two parental cell lines with distinct molecular profiles, HT-29 (chromosome instable, CIN) and HCT-116 (microsatellite instable, MSI).

We here report that chronic chemotherapeutic stress drives the evolution of “stemness” in CRC cells in a complex manner which is relevant for the elaboration of future therapeutic strategies. In addition, our results reveal that classical WNT-signaling inhibitors have toxic off-target activities that need to be addressed for their clinical development.

## RESULTS

### Chronic chemotherapeutic stress is accompanied by altered CD44 splicing

CD44 is a broadly distributed multifunctional glycoprotein adhesion molecule that is widely used as a stem cell marker [18]. Through alternative splicing, cells can produce a large family of CD44 protein isoforms which are involved in a variety of biological processes. Normal epithelial cells in the colon express the standard form of CD44 (CD44s) whereas adenomas, carcinomas and CRC metastasis may also express CD44 variants (CD44v) containing additional exons that are coding for insertions in the membrane-proximal extracellular region [19].

**Figure 1:**
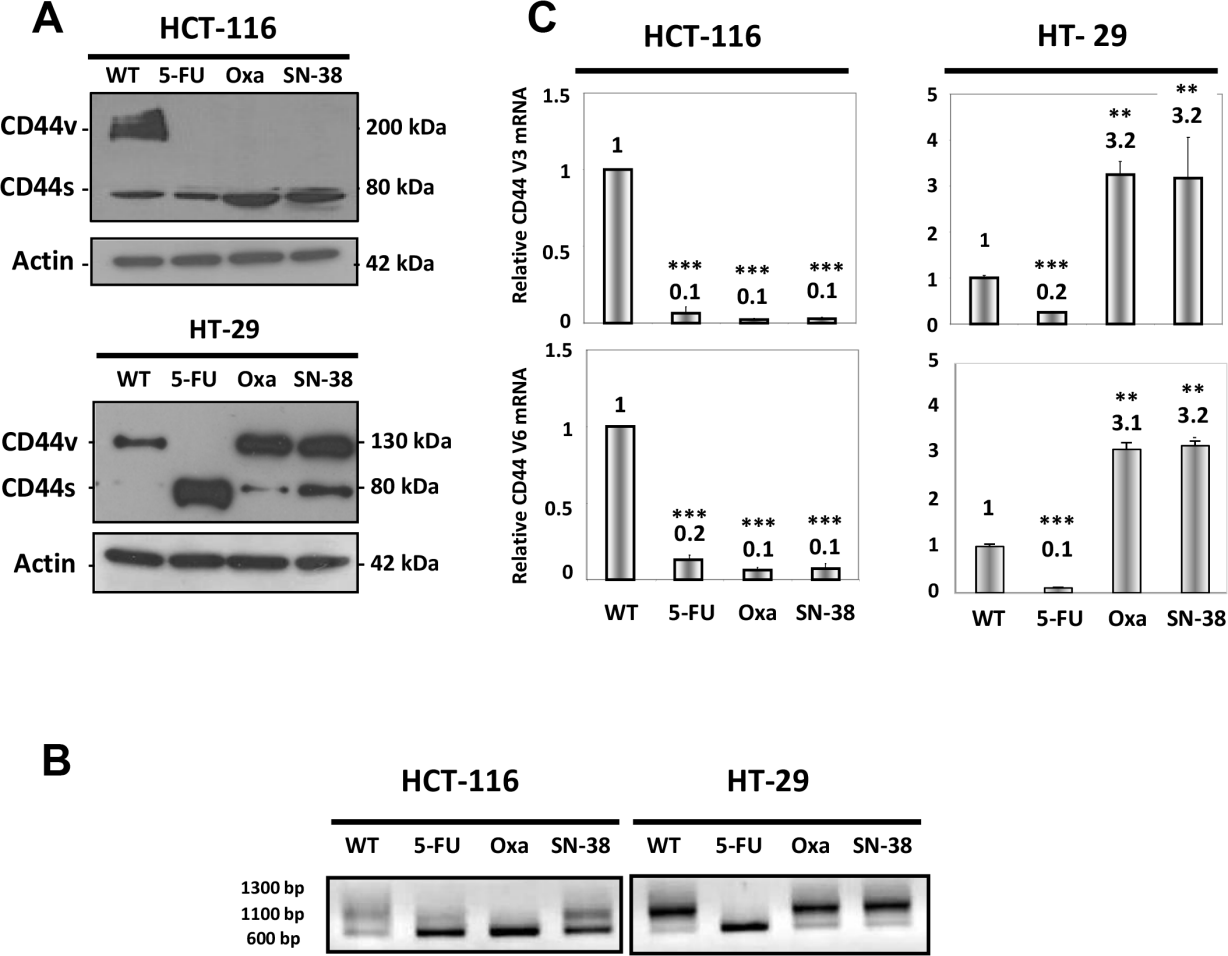
CD44 expression in parental and drug-resistant CRC cells **A.** Western blot analysis of CD44 expression in parental and drug-resistant HCT-116 (upper panel) or HT-29 cells (lower panel) using an antibody that detects all CD44 isoforms. CD44s, standard form of CD44; CD44v, variant forms of CD44. Actin was used as loading control. **B.** RNA was extracted from parental and drug-resistant cells and analyzed by qRT-PCR using primers that detect all isoforms of CD44 followed by agarose gel electrophoresis. **C.** RNA was extracted from parental and drug-resistant cells and analyzed by qRT-PCR using primers that specifically detect the CD44v3 or CD44v6 isoforms. Data were derived from three independent experiments. Columns correspond to the relative mRNA expression of the indicated mRNA normalized to GAPDH. Bars, SD. **p < 0.01; ***p < 0.001 as determined by Student's *t*-test.

Western blot analysis revealed that the parental HCT-116 cells predominantly expressed CD44v isoforms in contrast to the three drug-resistant HCT-116 variants that almost exclusively expressed CD44s (Figure 1A upper panel). Parental HT-29 cells expressed exclusively CD44v isoforms while the oxaliplatin- and SN38-resistant variants showed increased expression of CD44v as well as expression of CD44s. In comparison, HT-29/5-FU cells expressed exclusively CD44s (Figure 1A lower panel). To confirm that the altered size distribution was linked to alternative splicing, rather than to potential differences in glycosylation [20], CD44 mRNA was characterized further by qRT-PCR followed by agarose gel electrophoresis (Figure 1B). The results confirm the differences in size between CD44 transcripts.

We then focused on CD44 variants containing exons 3 (CD44v3) and 6 (CD44v6). The CD44v3 isoform undergoes heparin sulphate modification and is able to interact with various growth factors including heparin-binding epidermal growth factor and basic fibroblast growth factor [21, 22], whereas the CD44v6 isoform has been shown to act as a co-receptor for the receptor tyrosine kinase c-MET [23]. In agreement with the Western blot analysis, quantitative RT-PCR analysis of CD44 mRNA using isoform-specific primers for v3 and v6 showed decreased expression of CD44v3 and CD44v6 in the three drug-resistant HCT-116 cells as well as for the HT-29/5-FU cells (Figure 1C). In contrast, increased expression (about 3-fold) was observed for both CD44v3 and CD44v6 in the HT-29/Oxa and HT-29/SN38 cells.

### Differential influence of chronic chemotherapeutic stress on the expression of CD133, CD166 and Lgr5

**Figure 2:**
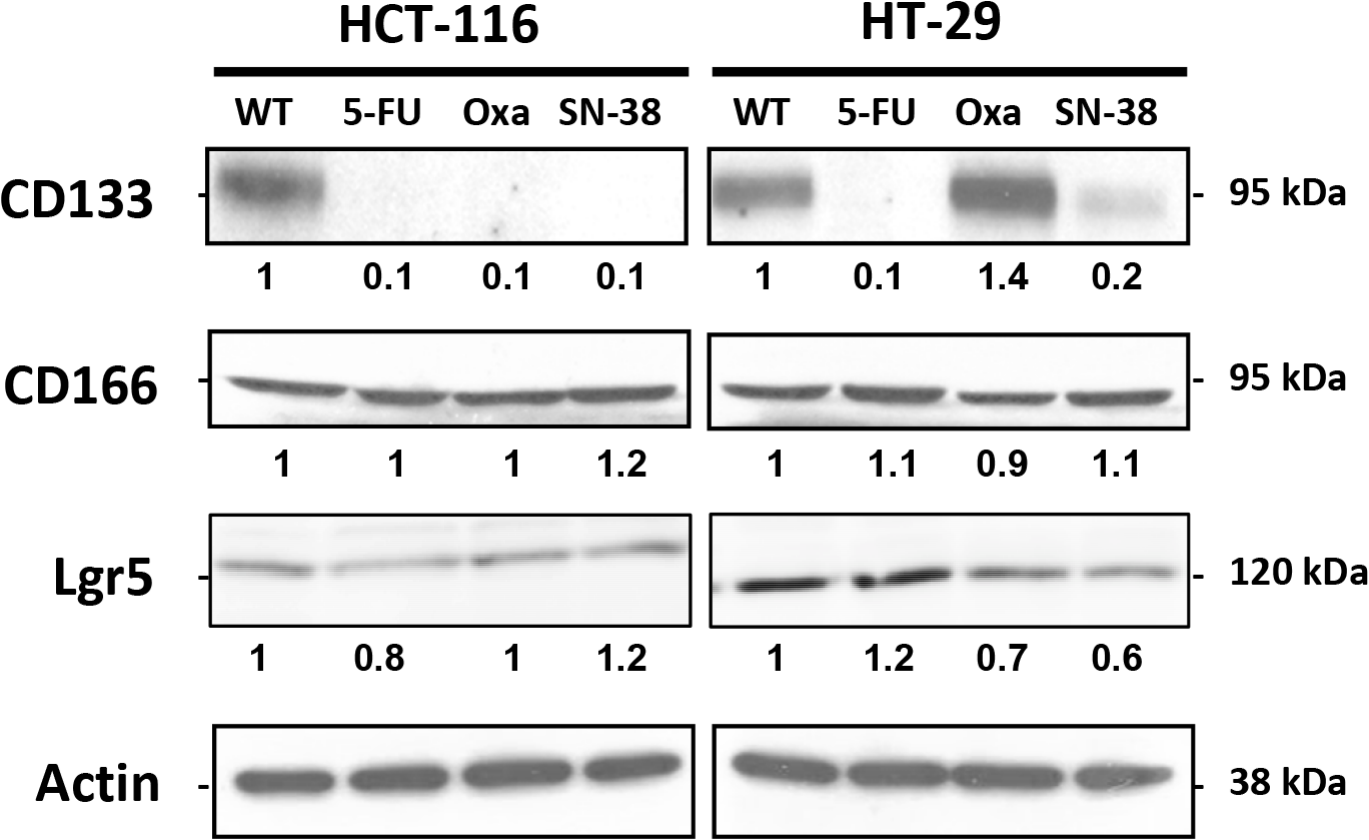
Expression of CD133, CD166 and Lgr5 in parental and drug-resistant HCT-116 and HT-29 cells as determined by Western blot analysis Actin was used as loading control. Relative protein levels were calculated by densitometry and are listed below the bands.

CD133 is a widely used stem cell marker [24] whereas CD166 and Lgr5 are expressed on the surface of epithelial cells within the colon stem cell niche [25, 26]. Western blot analysis show that CD133 protein was strongly expressed by the parental HCT-116 and HT-29 cells as well as by the HT-29/Oxa cells (Figure 2) while CD133 expression was weak in the HT-29/SN-38 cells and marginal in drug-resistant HCT-116 variants and HT-29/5-FU cells. In clear contrast, Western blot analysis revealed no alteration in the expression of CD166 among the eight cell lines (Figure 2). Lgr5 expression was comparable for HT-29/5-FU cells and slightly downregulated for HT-29/Oxa and HT-29/SN38 cells, compared to the parental cells, whereas Lgr5 expression was marginal for all four HCT-116 cell lines. Therefore, chronic drug exposure had divergent influence on the expression of the three stem cell markers indicating that their expression is not coordinated in CRC cell lines.

### Chronic chemotherapeutic stress is accompanied by altered aldehyde dehydrogenase (ALDH) activity

**Figure 3:**
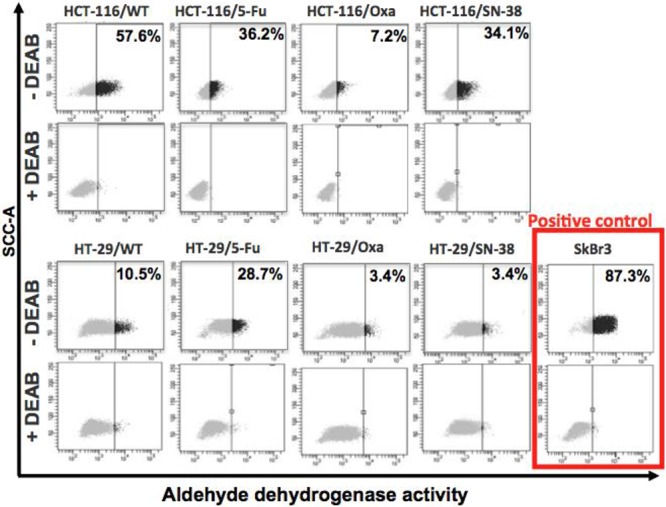
Flow cytometry analysis of aldehyde dehydrogenase (ALDH) activity Parental and drug-resistant HCT-116 and HT-29 cells were treated with ALDEFLUOR in the absence or presence of the ALDH inhibitor DEAB in order to establish the baseline fluorescence of the cells thereby defining the ALDEFLUOR positive region. The numbers indicate the fraction of cells present in the ALDH positive region.

ALDH is considered as a biomarker for the stem cell/progenitor cell phenotype [27, 28]. Comparison of the ALDH enzymatic activity by the ALDEFLUOR assay in the parental and resistant cell lines revealed a clear decrease in ALDH activity for all three HCT-116 drug-resistant variants that was particularly pronounced for the HCT-116/Oxa cells. The ALDH activity was also decreased for the HT-29/Oxa and HT-29/SN-38 cells, in contrast to the HT-29/5-FU cells where the ALDH activity was increased (Figure 3). Therefore, most of the drug-resistant CRC cells showed decreased ALDH activity whereas ALDH was upregulated in the HT-29/5-FU cells.

### Chronic chemotherapeutic stress may be accompanied by loss of colonosphere capacity

**Figure 4:**
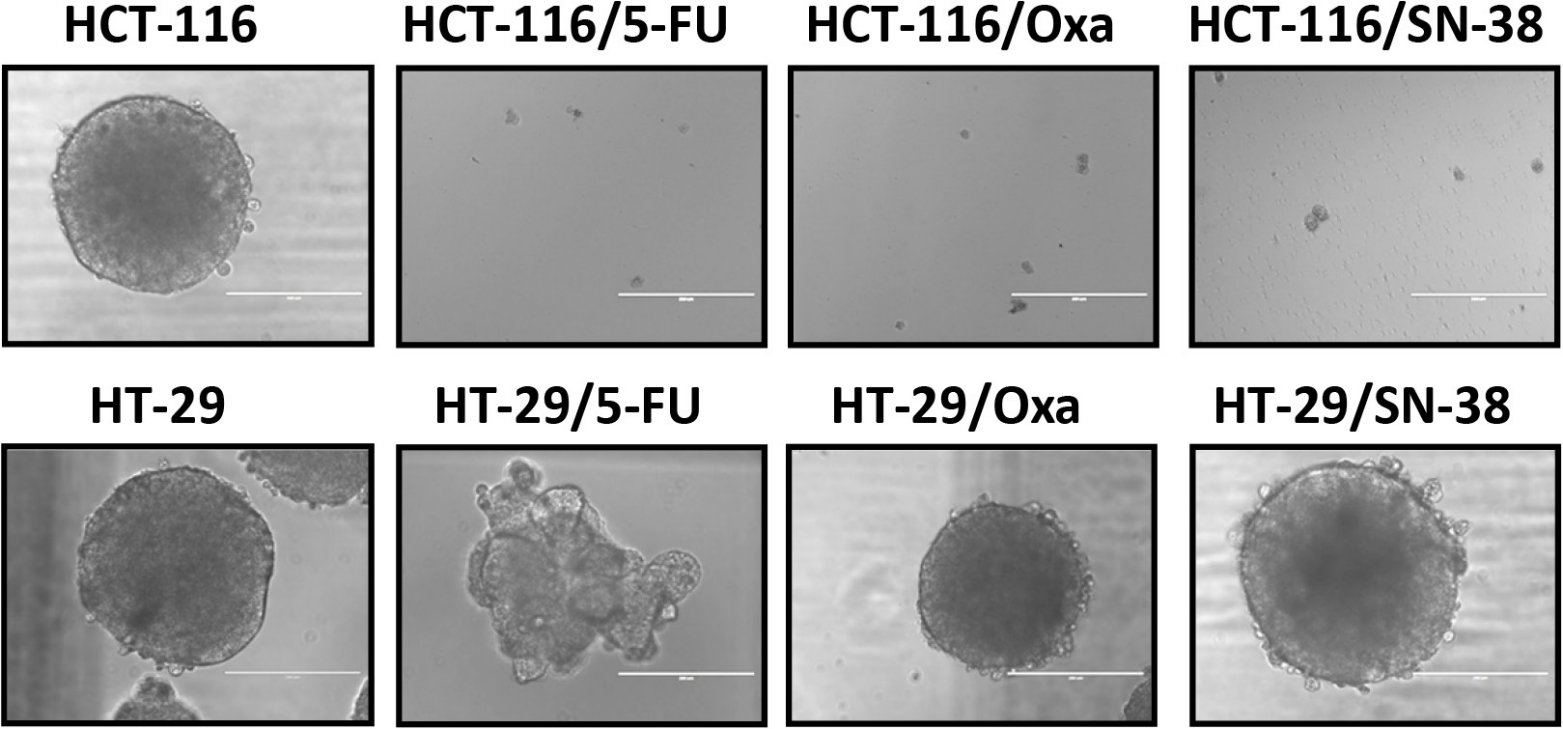
Colonosphere formation Parental and drug-resistant HCT-116 and HT-29 cells were plated in serum-free non-adherent culture conditions. The images are representative of colonospheres formation after 14 days in culture.

Colon stem cells as well as some colon cancer cells are able to form colonospheres in serum-free media supplemented with growth factors, when plated in limited numbers under anchorage-independent conditions [29]. Both HCT-116 and HT-29 parental cells were able to form uniform and regular colonospheres under these conditions (Figure 4).

Unexpectedly, the capacity to form colonospheres was lost for all three drug-resistant HCT-116 variants. In comparison, HT-29/Oxa and HT-29/SN-38 cells had retained the capacity to form colonospheres with a morphology comparable to the parental cells whereas the 5-FU resistant HT-29 cells formed loose irregular aggregates with structural similarity to the spheroids previously described for MDA MB-231 [30] and MCF7-sh-WISP2 cells [31].

### Chronic chemotherapeutic stress has different influence on the capacity to efflux Hoechst 33342 dye

**Figure 5:**
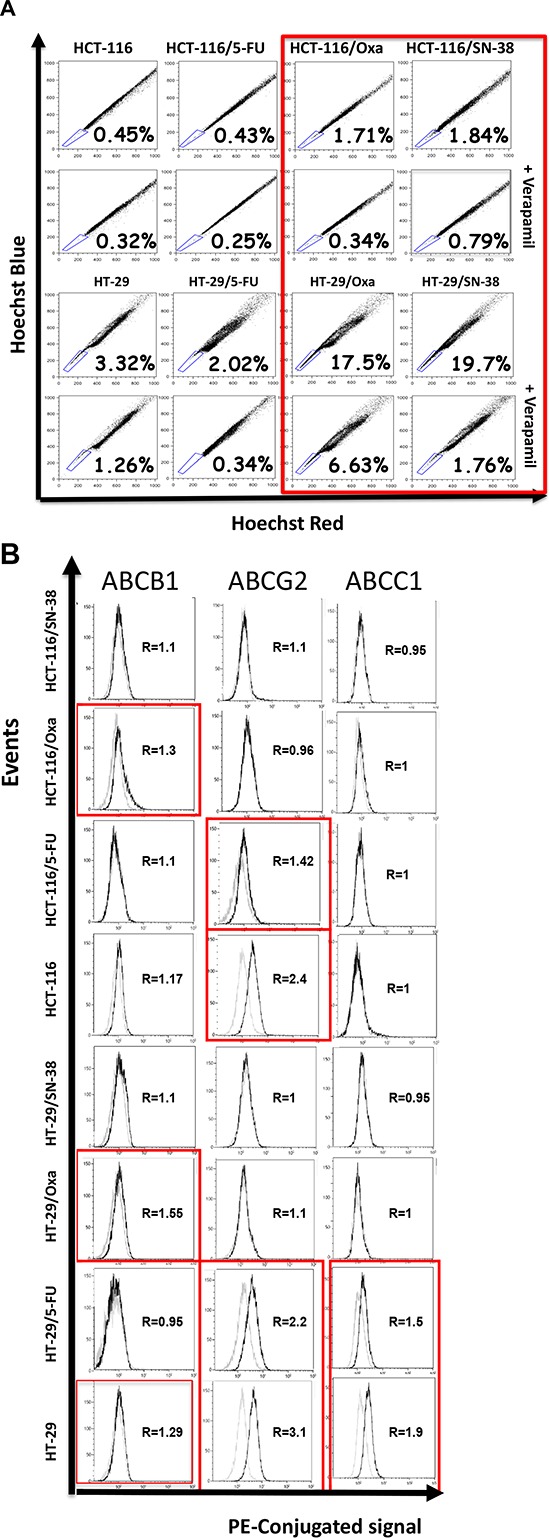
A. Accumulation of Hoechst 33342 as determined by flow cytometry analysis Parental and drug-resistant HCT-116 and HT-29 cells were exposed to Hoechst 33342 in the absence or presence of verapamil, a non-specific inhibitor of ABC membrane transporters, and Hoechst blue and Hoechst red fluorescence were measured by flow cytometry. The numbers indicate the fraction of the side population (outlined in the lower left corner) and are representative of three independent experiments. **B.** Expression of ABCB1 (PGP, MDR1), ABCG2 (MXR, BCRP) and ABCC1 (MRP1) as determined by flow cytometry analysis. Parental and drug-resistant HCT-116 and HT-29 cells were incubated with phycoerythricine-conjugated antibodies (solid black lines) or the corresponding isotype antibody (thin black lines) to determine the baseline fluorescence. R corresponds to the ratio of the mean intensities and is representative of two independent experiments.

A characteristic shared by many adult stem cells as well as some chemo-naive tumor cells is the ability to efflux Hoechst due to elevated expression of ABC transporters on the cell surface [32, 33]. The ability to extrude Hoechst 33342 dye was determined by flow cytometry analysis (Figure 5A) where the efflux capacity is considered as the difference in the proportion of cells present in the side population (SP, outlined in the lower left corner) in the absence and presence of the non-specific ABC transporter inhibitor verapamil. The results indicate modest modifications of the side population for the two 5-FU-resistant cell lines, compared to the corresponding parental cells. In contrast, the side population was increased for oxaliplatin- and SN38-resistant cells as outlined in red, which is particularly marked for the two HT-29 variants.

We then determined the relative expression of three drug transporters expressed by SP cells: ABCB1 (PGP, MDR1), ABCG2 (MRX, BCRP) and ABCC1 (MRP1) [34] by flow cytometry analysis. The results show that the expression of ABCB1, ABCG2, ABCC1 alone or in combination was increased for the oxaliplatin- and SN-38-resistant variants, whereas the two 5-FU-resistant cell lines show increased expression of ABCB1 (Figure 5B).

### The expression of stem cell markers and chemoresistance is not causally linked

To explore the link between chemoresistance and the expression of stem cell markers, the six resistant variants were maintained in drug-free media for a prolonged period of time. After more than 6 months (> 50 passages) we observed no detectable differences in the expression of stem cell markers for any of the resistant cell lines (data not shown) whereas the levels of drug resistance decreased markedly during this interval (Table 1). This was particularly striking for the HT-29/5-FU cells where the resistance ratio decreased from 12 to 2. Therefore, although the chemotherapeutic stress was driving the evolution of both the drug resistance and the altered expression of molecular and functional stem cell markers, the two phenotypes do not appear to be causally linked.

**Table 1:** Characteristics of parental and drug resistant cell lines used in this study

	Cells resistant to	Resistance level^a^	Resistance level^b^
HCT-116	Parental cells	1	1
HCT-116/5-FU	5-fluorouracil	12	7
HCT-116/Oxa	Oxaliplatin	15	6
HCT-116/SN-38	SN-38 (irinotecan)	10	6
HT-29	Parental cells	1	1
HT-29/5-FU	5-fluorouracil	12	2
HT-29/Oxa	Oxaliplatin	10	4
HT-29/SN-38	SN-38 (irinotecan)	5	3

The resistance level corresponds to the IC_50_ value for the resistant cells/IC_50_ value for the corresponding parental cells

a Resistance levels observed when cells are under continuous drug exposure

b Resistance levels observed when the resistant cells were maintained in drug-free media for 6 months

### Chronic chemotherapeutic stress modulates WNT-beta-catenin signaling

**Figure 6:**
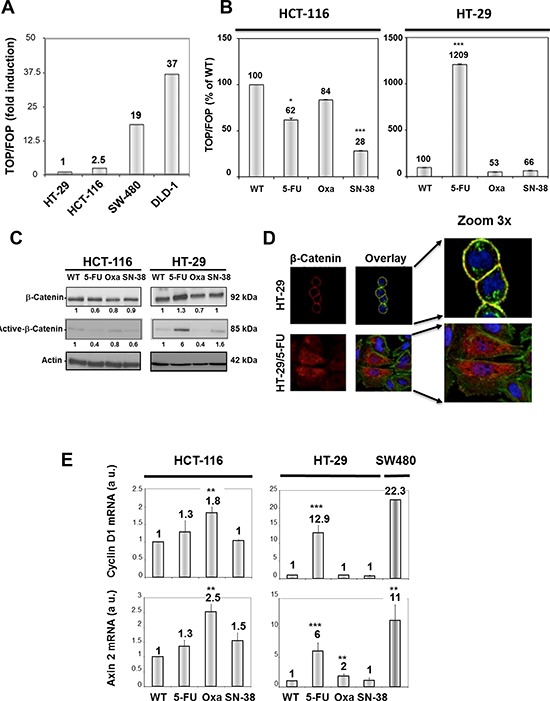
A. Comparison of WNT-signaling in HT-29 and HCT-116 cells in comparison with DLD-1 and SW480 cells WNT pathway activation was determined after transfection with Top-Flash and Fop-Flash plasmid constructs and is expressed in comparison with the TOP/FOP luciferase activity in HT-29 cells. **B.** WNT-signaling in parental and resistant HCT-116 (left) and HT-29 cells (right) expressed as % of control. Bars, SD. *p < 0.05; **p < 0.01; ***p < 0.001 as determined by Student's *t*-test. **C.** Expression of total and unphosphorylated beta-catenin in parental and drug-resistant variants as determined by Western blot analysis. Actin was used as loading control. Relative protein levels were calculated by densitometry and are listed below the bands. **D.** Fluorescence microscopy of beta-catenin localization in HT-29 and HT-29/5-FU cells as determined by immunostaining with a beta-catenin-directed antibody (red). Nuclei were stained by DAPI (blue) while actin was revealed by phalloidin (green). The overlay outlines the localization of beta-catenin on the cytoplasmic membrane (yellow) in the parental, but not in the 5-FU resistant cells. **E.** Expression of Cyclin D and Axin 2 mRNA as determined by qRT-PCR. Data were derived from three independent experiments. Columns, relative mRNA expression of the indicated mRNA normalized to GAPDH. Bars, SD. *p < 0.05; **p < 0.01; ***p < 0.001 as determined by Student's *t*-test.

WNT pathway activation was determined after transfection with TOP-Flash and FOP-Flash plasmid constructs and is expressed in arbitrary units as the ratio of the TOP/FOP luciferase activity. First, we evaluated WNT-signaling for the two parental cell lines in comparison with two reference cell lines, DLD1 and SW480 that show strong intrinsic WNT-signaling activity [35, 36]. The results (Figure 6A) indicate modest WNT-signaling activity for both HT-29 and HCT-116 parental cell lines compared to DLD1 and SW480 cells. Next, WNT-signaling was compared between parental and drug-resistant variants. The results (Figure 6B) reveal that the WNT-signaling activity was decreased for the three HCT-116 variants which was particularly marked for the SN-38-resistant cells. WNT-signaling activity was also downregulated in HT-29/Oxa and HT-29/SN-38 cells, compared to the parental cells. In marked contrast, WNT-signaling activity was upregulated 12-fold in the HT-29/5-FU cells.

Activation of the canonical WNT pathway is generally associated with nuclear accumulation of active, dephosphorylated β-catenin that has escaped proteasome degradation. In agreement with the findings shown in Figures 6A and 6B, Western blot analysis revealed a 6-fold increase in the expression of the active, unphosphorylated form of beta-catenin in HT-29/5-FU cells whereas the expression was either decreased or unchanged for the other drug-resistant variants (Figure 6C).

The localization of beta-catenin in parental and 5-FU resistant HT-29 cells was characterized by fluorescence microscopy (Figure 6D). The results indicate that beta-catenin (indicated in red) is predominantly membrane-associated in the parental HT-29 cells but displays a cytoplasmic/nuclear staining in the HT-29/5-FU cells. Nuclear accumulation of beta-catenin allows complex formation with the transcription factor T cell factor/Lymphoid enhancer factor (TCF/LEF) thereby leading to transcriptional activation of target genes like Cyclin D1 [37] and Axin 2 [38]. In agreement, the levels of Cyclin D1 and Axin 2 mRNA, as determined by qRT-PCR, were increased in the HT-29/5-FU cells but not in the other drug-resistant variants (Figure 6E). Unexpectedly, a modest, but reproducible increase in mRNA levels for cyclin D1 and Axin 2 was observed for the oxaliplatin-resistant HCT-116 cells while the 5-FU and SN-38 resistant HCT-116 cells were comparable with the parental HCT-116 cells.

### The WNT-signaling inhibitors ICG-001 and PKF 118 downregulate WNT-activity without any selective cytotoxic effects

**Figure 7:**
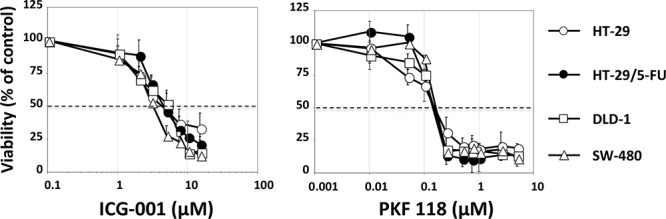
Viability of HT-29, HT-29/5-FU, DLD1 and SW480 cells after 120 hours continued exposure to ICG-001 (left) or PKF 118 (right) followed by MTT determination Bars indicate SD and are shown when they exceed symbol size. The data represents at least three independent experiments each done in duplicate.

To establish if the strong WNT activity in HT-29/5-FU cells was accompanied by increased sensitivity to WNT inhibition, we selected two WNT-signaling inhibitors with different mechanism of action. ICG-001 is a selective low molecular-weight inhibitor, which antagonize β-catenin/TCF-mediated transcription by specifically binding to CBP (cyclic AMP response element-binding protein), thereby disrupting the interaction between CBP and β-catenin [39]. PKF 118–310 binds selectively to beta-catenin, thereby disrupting its interaction with TCF-4 [40]. The growth inhibitory effects of ICG-001 and PKF 118 were determined by the MTT viability assay after 120 hours continuous drug exposure [41] and is expressed as IC_50_ values (drug concentration inhibiting cell growth by 50% compared to untreated controls). The IC_50_ values were around 3 μM for ICG-001 and 0.3 μM for PKF 118 (Figure 7) in agreement with previous findings [39, 40]. However, there was no selective cytotoxicity toward either HT-29 or HT-29/5-FU cells. Similar experiments with DLD-1 and SW480 cells that are both chemo-naïve and show high intrinsic WNT-signaling (Figure 6) resulted in similar IC_50_ values for both ICG-001 and PKF 118 (Figure 7), and this although the 4 cell lines tested differ almost 40-fold with respect to Wnt/beta catenin activity (Figure 6A).

To confirm these unexpected findings, the cytotoxic activities of ICG-001 and PKF 118 toward HT-29, HT-29/5-FU, SW480 and DLD-1 cells were determined by the highly sensitive colony formation assay. The results (SI Figure 1) show that the cytotoxic activity of PKF 118 was comparable between the 4 cell lines. For ICG-001, there was up to 3-fold differences in the cytotoxic activity (IC_50_ values) between the most sensitive and the most resistant cell line, but without any obvious correlation between IC_50_ value and the WNT/beta-catenin activity.

To establish if the increased WNT/beta-catenin activity in the HT-29/5-FU cells was causally related to 5-FU resistance, we determined the influence of 5-FU on colony formation in the absence or presence of PKF 118 or ICG-001. The results (SI Figure 2) show no influence of PKF 118 or ICG-001 on the cytotoxic activity of 5-FU in neither HT-29 nor HT-29/5-FU cells.

**Figure 8:**
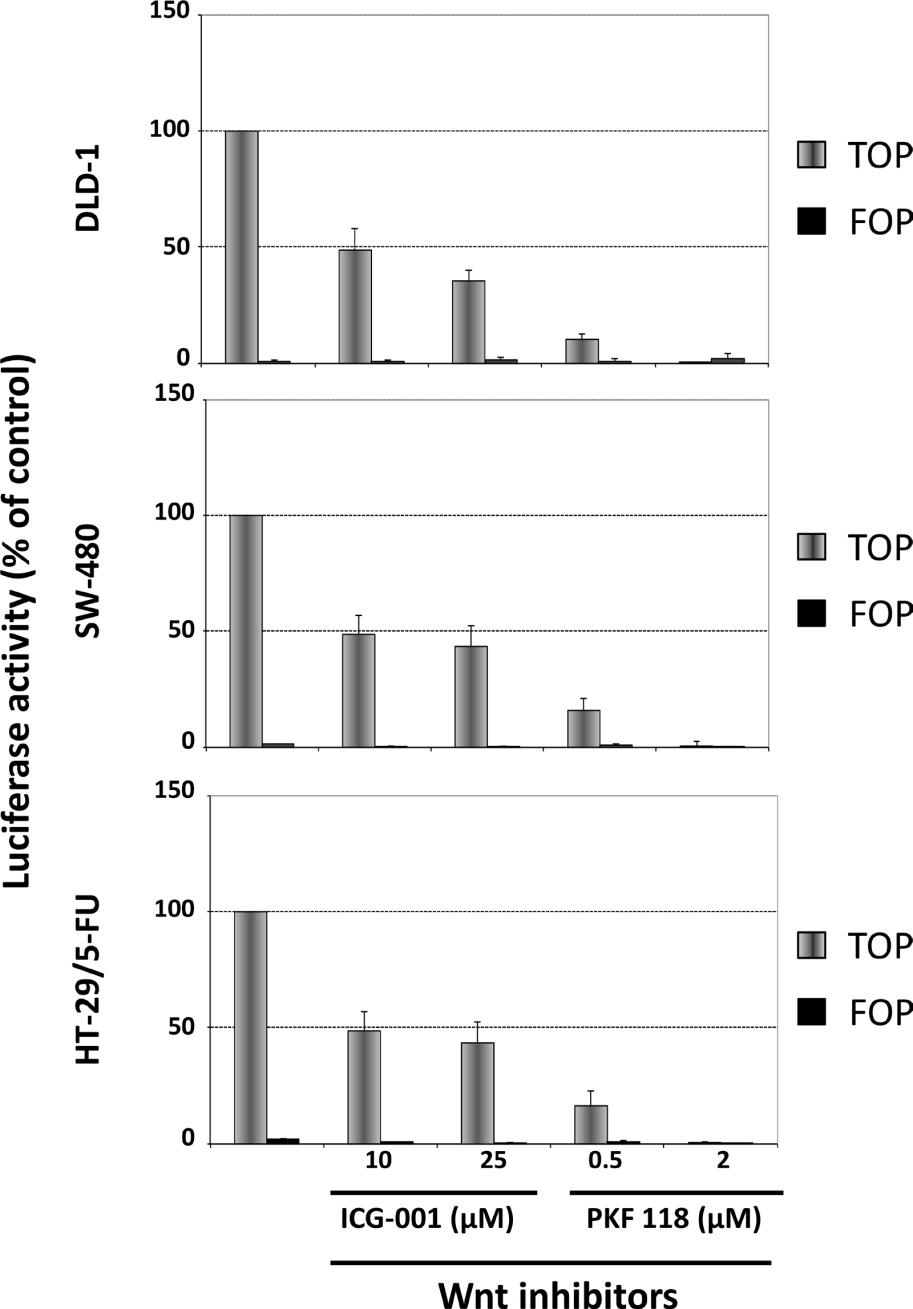
Influence of ICG-001 and PKF 118 on WNT-signaling in DLD-1 cells (top), SW-480 cells (middle) or HT-29/5-FU cells (bottom) as determined by the luciferase activity after transfection with Top-Flash or Fop-Flash plasmid constructs Bars, SD. The data represents two independent experiments each done in duplicate.

We then characterized the influence of ICG-001 and PKF 118 on WNT-signaling in DLD-1, SW480 and HT-29/5-FU cells after transfection with TOP-Flash or FOP-Flash plasmid constructs. The results show that 24 hours exposure to ICG-001 and PKF 118 is associated with a clear decrease in WNT-signaling (Figure 8).

Taken together, these results show that although the two WNT signaling inhibitors do indeed inhibit WNT-signaling within a relevant dose range, this is not accompanied by any selective cytotoxic effects toward cells with high levels of WNT signaling.

## DISCUSSION

Most solid tumors contain a subfraction of cells with molecular and functional characteristics of stem/progenitor cells that show intrinsic chemoresistance [16, 42, 43]. This suggests that chronic chemotherapeutic stress may provide a selective advantage to cells with a high degree of “stemness”. To explore the link between chronic chemotherapeutic stress and “stemness”, we carried out a comprehensive molecular and functional analysis of six independently selected CRC cell lines with acquired resistance to three chemotherapeutic agents with different mechanisms of action (5-fluorouracil, oxaliplatin and irinotecan) derived from two parental cell lines with distinct molecular profiles, HT-29 (chromosome instable, CIN) and HCT-116 (microsatellite instable, MSI). Since the expression of stem-cell markers may not be regulated in a coordinated fashion, we used a battery of four widely used stem-cell/progenitor markers (CD44, CD133, CD166 and Lgr5) and three functional tests (aldehyde dehydrogenase activity, colonosphere formation and exclusion of Hoechst dye).

Our results show that although chronic chemotherapeutic stress may indeed be accompanied by the appearance of cells with increased stemness, the outcome is more complex than originally proposed. One cell line, HT-29/5-FU showed a clear increase in aldehyde dehydrogenase activity, increased expression of the ABC transporter ABCB1 and formed loose cellular aggregates in serum-free media under low attachment, in contrast to the parental HT-29 cells that formed regular spheroids under the same conditions. Therefore, chronic chemotherapeutic stress was accompanied by increased “stemness” in these cells.

In contrast, all three drug-resistant HCT-116 variants had lost CD133 expression, displayed decreased aldehyde dehydrogenase activity and had lost the capacity to form spheroids, suggesting that these cells had either differentiated further or, alternatively, had undergone trans-differentiation, most likely via EMT (epithelial-mesenchymal transition). The latter possibility is supported by a switch in CD44 isoform expression from a mixture of CD44v and CD44s isoforms in the parental cells to CD44s in all three HCT-116 variants, since the expression of CD44s has been causally linked to the EMT phenotype in different tumor types including the colon [44–47]. Interestingly, the HT-29/5-FU cells have undergone a similar CD44 switch, as shown by strong expression of the CD44s isoform. In addition, these cells display an altered morphology, consistent with a mesenchymal phenotype, as illustrated in Figure 6D.

Besides CD44 isoform switching, the three HCT-116 variants and HT-29/5-FU cells have lost the expression of CD133. Although CD133 is widely used as an universal stem-cell marker, recent results suggest that this might not be applicable for the colon where CD133 is ubiquitously expressed on differentiated colonic epithelium in both adult mice and in humans [48]. Interestingly, the same study reported that CD133^+^ CRC cells form less aggressive tumors, which is supported by recent clinical studies where strong CD133 expression, as determined by immunohistochemistry, is associated with longer progression-free survival in patients with metastatic CRC [49, 50]. Assuming that these four resistant cells lines have indeed undergone EMT, this would mean that EMT can be associated (HT-29/5-FU) or not (HCT-116/5-FU, HCT-116/Oxa, HCT-116/SN-38) with “stemness”, in agreement with recent models [51, 52].

Finally, two drug-selected cell lines, HT-29/Oxa and HT-29/SN-38 show a heterogeneous response with respect to stem cell markers in combination with the presence of a prominent side population linked to increased expression of ABCB1, ABCG2 and ABCC1. Therefore, it appears that prolonged chemotherapeutic stress was principally associated with upregulation of ABC drug transporters in these two cell lines.

Interruption of chronic drug exposure was accompanied by diminished drug resistance in agreement with the clinical experience, where drug-free intervals are typically associated with restoration of drug-sensitivity. In contrast, the altered expression of stem cell markers appears more permanent. This observation is coherent with the dynamic nature of tumor cells that evolves under chemotherapeutic stress and provides interesting therapeutic perspectives for second-line treatment. For example, we recently reported that acquired irinotecan resistance was associated with upregulation of EGFR- and SRC-signaling pathways that can be targeted with selective anticancer agents [15].

The WNT/beta-catenin signaling pathway is altered in more than 90% of patients with CRC [2] as well as in patients with breast cancer [53, 54] and hepatocellular carcinoma [55] making it a particularly attractive therapeutic target. However, the pathway presents several features that suggest that it may also be a difficult target. For example, WNT-signaling appears to play a much broader role not only in crypt epithelial cells but also in differentiated epithelial and mesenchymal cells of the small intestine and colon than was previously anticipated [56]. This is supported by recent findings reporting that epithelial WNTs are dispensable and that stromal production of WNTs can fully support normal murine intestinal homeostasis [57]. A comprehensive analysis of human tumor samples from patients with colon or rectal cancer revealed that multiple lesions affecting the WNT signaling pathway may co-exist in the same tumor suggestive of a complex signaling network [2]. Finally, it was shown that the canonical WNT suppressor Axin 2 promotes colon carcinoma oncogenic activity rather than functioning as a tumor suppressor as expected [58].

Our results show that WNT-signaling activity is upregulated in the HT-29/5-FU cells as shown by a luciferase reporter assay, the expression and localization of beta-catenin as well as by transcriptional upregulation of Cyclin D1 and Axin 2, two prominent transcriptional targets of canonical WNT-signaling. Although WNT-signaling could be attenuated in the HT-29/5-FU cells by the WNT-signaling inhibitors ICG-001 and PKF 118, this was not accompanied by any selective growth inhibitory effects suggesting that the cytotoxic activity of these compounds is not directly linked to WNT-signaling inhibition. This unexpected finding was subsequently confirmed in two chemo-naïve CRC cell lines with strong WNT-signaling. It is possible that the cytotoxic effects of WNT inhibition may be context-dependent and rely of the strength of additional signaling pathways like MAP kinase signaling [59] or VEGFR1 signaling [60]. However, although the 24 hours drug exposure used for the reporter assay and the 120 hours drug exposure used for the MTT viability assay are not directly comparable, it is noticeable that the drug concentrations needed for WNT-signaling inhibition and for the cytotoxic effects are within the same dose range. This suggests that both inhibitors influence additional targets (off-targets) within the same dose range as WNT-signaling inhibition that can induce cell death independent of the WNT pathway. Future development of WNT-signaling inhibitors will depend on identification of these off-targets in order to obtain WNT inhibitors with increased selectivity.

Taken together, we here show that chronic chemotherapeutic stress drives the evolution of “stemness” in a heterogeneous, context-dependent manner in CRC cells which may provide clues for future therapeutic strategies for second-line treatment. Furthermore, our findings reveal that the cytotoxic activities of WNT-signaling inhibitors do not depend on WNT-signaling as such but rather on off-targets that remain to be identified for further development of this category of molecules.

## MATERIALS AND METHODS

### Cell culture

DLD-1 cells were kindly provided by Richard Hamelin (Paris, France) while SW-480 colon carcinoma cells were purchased from American Type Culture Collection (Rockville, MD). HT-29 cells were provided by Thécla Lesuffleur (Paris, France) and HCT-116 cells were a kind gift from Bert Vogelstein (Baltimore, MD).

The cells were maintained in, McCoy's A (HCT-116), DMEM (HT-29, SW480) or RPMI (DLD-1) supplemented with 5% fetal calf serum and 1% penicillin/streptomycin (PAA).

HT-29 or HCT-116 cells were exposed to increasing doses of 5-fluorouracil (Teva-Pharma), oxaliplatin (Eloxatin, Sanofi-Synthelabo) or SN-38 (the active metabolite of irinotecan, Abatra-technology) in the culture media as described in detail elsewhere [9, 10, 15]. The properties of parental and drug-resistant cells are detailed in Table 1.

### Reagents and antibodies

PFK118–310 was purchased from Sigma-Aldrich while ICG-001 was obtained from Selleck Chemicals. Alexa fluor 488 phalloidine and 4′, 6-diamidino-2-phenylindole (DAPI) were purchased from Sigma. The antibodies used for Western blot analysis and immunocytochemistry were: rabbit anti-CD44 (Abcam # 51037), rabbit anti-CD133 (Cell Signaling # 3663), mouse anti-CD166 (Abcam # 175422), rabbit anti-Lgr5 (Sigma-Aldrich # HPA012530), goat anti-actin (Santa Cruz # sc-1615), rabbit anti-beta-catenin (Cell Signaling # 8480), mouse antibodies specific for the active form of beta-catenin, dephosphorylated on Ser 37 and Thr 41 (Millipore, clone 8E7, # 05–665). Anti-rabbit IgG horseradish peroxidase-linked antibodies and anti-mouse IgG horseradish peroxidase-linked antibodies were from Cell Signaling whereas Cy3-conjugated anti-mouse secondary antibodies were obtained from Jackson ImmunoResearch Labs.

The following antibodies were used for flow cytometry analysis: phycoerythricine-conjugated mouse anti-human ABCB1 (BD Biosciences # 557003), phycoerythricine-conjugated mouse anti-human ABCG2 (BD Biosciences # 561180), mouse anti-human ABCC1 (BD Biosciences # 557594) and anti-mouse phycoerythricine-conjugated secondary antibodies (BD Biosciences # 555574).

### Viability assay

The growth inhibitory effects were determined by the MTT (3-(4, 5-dimethyl-thiazol-2yl)-2, 5-diphenyl-tetrazolium bromide) viability test as previously described [41] with minor modifications. Cells (7,000 cells per well for HCT-116, 10,000 per well for HT-29, SW480 and DLD-1) were seeded in 24-well plates in culture media containing 5% FCS, incubated in drug-free media for 24 hours followed by drug exposure for 120 hours. Cellular viability was determined by exposing cells to the MTT tetrazolium salt for 4 hours at 37°C and the formation of formazan was measured at 590 nm by a microplate reader. The IC_50_ value is defined as the drug concentration causing \50% reduction of viable cells compared to the untreated control cells. All values are averages of at least 3 independent experiments each done in duplicate.

### Western blot analysis

Western blot analysis was carried out as described previously [61, 62] with minor modifications. Cells were harvested at 70% confluence and cellular lysates were prepared in RIPA buffer (150 mM NaCl, 50 mM Tris pH 7.4, 0.1% SDS, 1% NP-40, 2 mM EDTA, 1 mM Na_3_VO_4_, 1 mM PMSF, 1 mM Na-F) supplemented with protease inhibitors (Roche) according to the manufacturer's instruction. Proteins were resolved on SDS-PAGE gels (8–12% acrylamide) in a denaturing buffer followed by transfer to nitrocellulose membranes. Membranes were incubated with anti-CD44, anti-CD133, anti-CD166, anti-Lgr5, anti-beta-catenin, anti-active beta-catenin or anti-actin antibodies followed by incubation with the appropriate secondary horseradish peroxidase-conjugated antibodies. Protein expression was revealed with enhanced chemiluminescence reagents (ECL Amersham).

### qRT-PCR

Total RNA was extracted from parental and drug-resistant HCT-116 and HT-29 cells with MRC Tri-reagent, according to the manufacturer's instructions. RNA quantity and purity was determined by using a NanoDrop ND-1000 and total RNA from each sample was reverse transcribed using Revertaid H Minus First Strand cDNA Synthesis Kit (Thermo Scientific) followed by amplification of the DNA product with a SYBR Green kit (Promega). PCR primers were designed with the logiciel primer3 and gene expression was normalized to GAPDH as previously described [31].

The threshold was set above the non-template control background and within the linear phase of target gene amplification to calculate the number of cycles at which the transcript was detected. Gene expression values were calculated based on the comparative delta CT method [63] and normalized to the housekeeping gene GAPDH.

### Aldehyde dehydrogenase assay

The ALDFLUOR assay was performed according to the manufacturer's recommendations (StemCell Technologies). Briefly, cells were harvested with accutase (PAA), counted and rinsed with PBS buffer. Cells were re-suspended in the ALDFLUOR assay buffer and incubated with the ALDFLUOR substrate for 45 min at 37°C to allow substrate conversion. Then, half of the sample were transferred to tubes containing the specific ALDH inhibitor, diethylaminobenzaldehyde (DEAB). After centrifugation, cells were suspended in the ALDFLUOR assay buffer and the ALDH activity analysis was determined by flow cytometry at 493 nm using a FACS LSR II (BD Biosciences).

### Colonospheres formation assay

For colonosphere formation, cells (100 cells) were incubated in serum-free media composed of DMEM and F12 (1:1) in 96-well ultra-low-attachment plates (Corning). The media was supplemented with B27 (Life Technologies), 20 ng/ml EGF (Sigma-Aldrich), 10 ng/ml fibroblast growth factor (Sigma), and 100 Units/ml penicillin and 100 μg/ml streptomycin (PAA). The resulting colonospheres were examined using an Olympus microscope (10x) after 14 days and representative images collected from at least five microscopic fields.

### Side population (SP) assay

The side population was determined as previously described [64] with minor modifications. Cells were collected by accutase, pelleted and resuspended in culture media with 2% FCS, 10 mM Hepes pH7.4 and 100 Units/ml penicillin and 100 μg/ml streptomycin followed by labelling with Hoechst 33342 (Sigma-Aldrich) for 90 min 37°C, in the absence of presence of verapamil (Sigma-Aldrich). Then, cells were centrifuged and re-suspended in Hank's balanced saline solution (Life Technologies) containing 2% FCS and 10 mM Hepes. Cells were maintained in darkness at 4°C until flow cytometry analysis. The SP was determined by analysis of 10^6^ cells using a FACS LSR II (BD Biosciences) after counterstaining with propidium iodide to identify dead cells.

### Flow cytometry analysis

Cells were grown to 70% confluence, detached with accutase, washed in PBS and fixed in PBS-PFA 1% for 10 minutes. Then, cells were labeled with phycoerythricine-conjugated mouse anti-human ABCB1, phycoerythricine-conjugated mouse anti-human ABCG2 or mouse anti-human ABCC1 followed by phycoerythricine-conjugated secondary antibodies.

### Immunocytochemistry

Cells were grown on coverslips for 24 hours. After washing with PBS, cells were fixed with 4% (v/v) paraformaldehyde in PBS for 20 minutes, washed twice with PBS-0.1% Tween 20 and permeabilized with 0.5% Triton x100 for 15 minutes at room temperature. Then, cells were washed and blocked for 30 minutes in 0.5% bovine serum albumin in PBS and incubated with anti-beta-catenin antibodies. The coverslips were washed with PBS-0.5% Tween 20 and incubated with Cy3-conjugated secondary. Cells were washed, counterstained with DAPI, mounted with Vectashield (Vector Laboratories) and observed by microscopy. Fluorescent images were captured using an inverted microscope (Olympus CKX41) with a digital compact camera (Olympus camedia C4000) and a 60x objective.

### WNT pathway reporter assay

For the luciferase reporter gene assays, cells were seeded in 12-well plates at 150,000 cells/well and incubated in drug-free media for 24 hours. Cells were then transfected with TOP-Flash (plasmids containing 3 TCF binding sites) and/or their negative counterpart FOP-Flash (plasmids containing mutant, inactive TCF binding sites) both from Upstate Technologies using the Fugene HD reagent (Promega). After 24 hours, cells were treated, or not, with various concentrations of ICG-001 or PKF118–320. The next day, the luciferase activity was determined using the luciferase assay system (Promega). RSV β-galactosidase vector was used for co-transfection in order to normalize the luciferase activity against β-galactosidase activity. All assays were performed in triplicate.

### Statistical analysis

The statistical analysis of experimental data was performed using a Student's paired *t*-test, and the results are presented as mean + SD. Symbols: *p < 0.05; **p < 0.01; ***p < 0.001.

## SUPPLEMENTARY FIGURES


