# Supplementary material for: Chronic chemotherapeutic stress promotes evolution of stemness and WNT/beta-catenin signaling in colorectal cancer cells: implications for clinical use of WNT-signaling inhibitors
Source: Oncotarget. 2015 May 11;6(21):18518–33. doi: 10.18632/oncotarget.3934 (PMC4621907; doi:10.18632/oncotarget.3934)
Supplement: Supplementary file 1 [file oncotarget-06-18518-s001.pdf]

SUPPLEMENTARY FIGURES

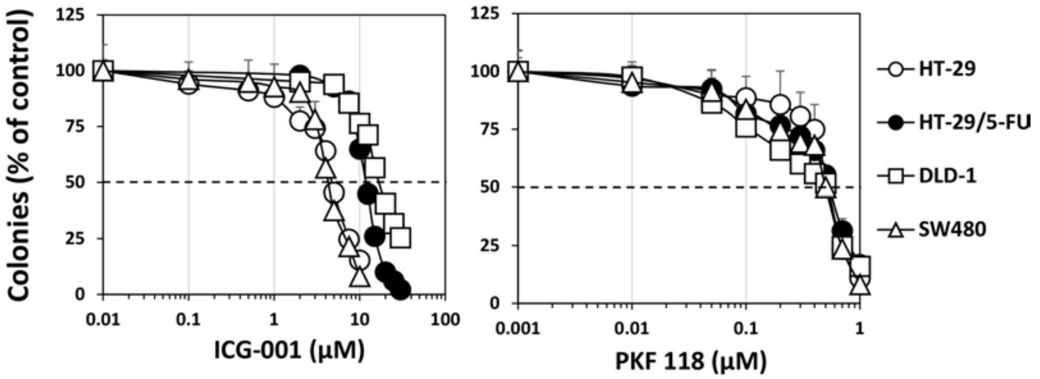

Supplementary Figure S1: Colony formation of HT-29, HT-29/5-FU, DLD1 and SW480 cells following continued exposure to ICG-001 (left) or PKF 118 (right). Bars indicate SD and are shown when they exceed symbol size. The data represent at least two independent experiments each done in duplicate.

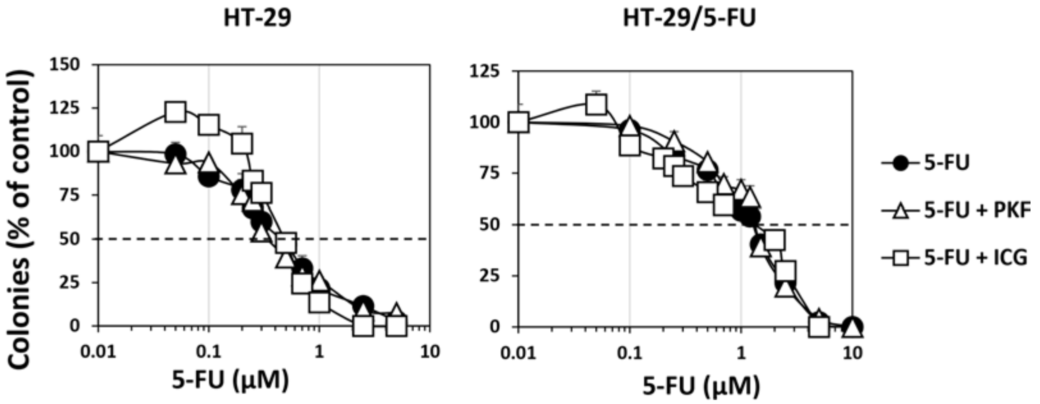

Supplementary Figure S2: Colony formation of HT-29 (left) and HT-29/5-FU (right) followed continued exposure to 5-FU in the absence or presence of ICG-011 or PKF 118. Bars indicate SD and are shown when they exceed symbol size. The data represent at least two independent experiments each done in duplicate.
